# Assessment of feasibility and acceptability of family-centered care implemented at a neonatal intensive care unit in India

**DOI:** 10.1186/s12887-021-02644-w

**Published:** 2021-04-13

**Authors:** Arti Maria, James A. Litch, Maria Stepanchak, Enisha Sarin, Rashmi Wadhwa, Harish Kumar

**Affiliations:** 1 Ram Manohar Lohia Hospital (RML) Hospital, New Delhi, India; 2 Global Alliance to Prevent Prematurity and Stillbirth (GAPPS), 19009 33rd Avenue W, Suite 200, Lynnwood, WA 98036 USA; 3 Vridhi Project, New Delhi, India

**Keywords:** Neonatal health, Newborn care, family-centered care, Family participatory care, child rights, newborn-parent unity, Neonatal intensive care unit, Special newborn care unit, India

## Abstract

**Background:**

A family-centered care (FCC) parent participation program that ensures an infant is not separated from parents against their will was developed for the caring of their small or sick newborn at a neonatal intensive care unit (NICU) in Delhi, India. Healthcare provider sensitization training directed at psychosocial and tangible support and an audio-visual training tool for parent-attendants were developed that included: 1) handwashing, infection prevention, protocol for entry; 2) developmentally supportive care, breastfeeding, expression of breastmilk and assisted feeding; 3) kangaroo mother care; and 4) preparation for discharge and care at home. The study aimed to examine the feasibility and acceptability of the FCC model in a NICU in India.

**Methods:**

A prospective cohort design collected quantitative data on each parent-attendant/infant dyad at enrollment, during the NICU stay, and at discharge. Feasibility of the FCC program was measured by assessing the participation of parent-attendants and healthcare providers, and whether training components were implemented as intended. Acceptability was measured by the proportion of parent-attendants who participated in the trainings and their ability to accurately complete program activities.

**Results:**

Of 395 NICU admissions during the study period, eligible participants included 333 parent-attendant/infant dyads, 24 doctors, and 21 nurses. Of the 1242 planned parent-attendant training sessions, 939 (75.6%) were held, indicating that program fidelity was high, and the majority of trainings were implemented as intended. While 50% of parent-attendants completed all 4 FCC training sessions, 95% completed sessions 1 and 2; 60% of the total participating parent-attendants completed session 3, and 75% completed session 4. Compliance rates were over 96% for 5 of 10 FCC parent-attendant activities, and 60 to 78% for the remaining 5 activities.

**Conclusions:**

FCC was feasible to implement in this setting and was acceptable to participating parent-attendants and healthcare providers. Parents participated in trainings conducted by NICU providers and engaged in essential care to their infants in the NICU. A standard care approach and behavior norms for healthcare providers directed psychosocial and tangible support to parent-attendants so that a child is not separated from his or her parents against their will while receiving advanced care in the NICU.

## Background

The first few weeks of an infant’s life are an important time for the formation of healthy attachment between newborn and parents. When an infant is born small or sick, however, separation from the parents during hospitalization can disrupt this bonding process [1]. Modern neonatal intensive care units (NICUs) rely on technically skilled staff to provide care to sick newborns and parental involvement is typically limited to brief visits despite evidence showing the importance of increased parent-infant interaction for newborn health, developmental outcomes, and parental stress [1, 2]. Most parents report anxiety, stress, and a lack of control during their NICU experience [3]. In low-resource settings, NICU care is further complicated by the limited availability of healthcare staff, resulting in high burden on healthcare providers and the potential for reduced quality of care for newborns [4]. Integrating parents into a newborn’s care during hospitalization can maintain newborn-parent unity, promote supportive developmental care, and facilitate stable newborn and parent attachment.

The family-centered care (FCC) approach was adapted from the Human Neonatal Care Initiative in the 1990s [5], and existing family participation in inpatient newborn care models to respond to these challenges [6–10]. FCC aims to develop and nurture the family’s role in partnership with the healthcare team in the care of a sick newborn [11, 12]. This approach has been shown to improve health and developmental outcomes in newborns, reduce parental stress, and improve healthcare provider satisfaction and resource allocation at health facilities [6–10]. FCC empowers, encourages, and supports the family as a caregiver, along with the nursing staff, to complement care for sick and small newborns. This approach creates an opportunity for parent–child bonding during the critical early life period and enhances parental caregiving competencies which may help parents to provide better care post-discharge. The philosophy of FCC has been recognized by multiple medical societies, the Institute of Medicine, healthcare systems, and Healthy People 2020 as an important strategy to improve patient health, satisfaction, and quality of care [13–16]. Recent studies showed a reduction of length of hospital stay without concomitant increase in readmission or return visits [17, 18]. Although implemented in multiple high-resource settings, no universal model of FCC exists. FCC focuses on the following principles: family participation in care, addressing family needs, collaboration, respect and dignity, and knowledge sharing between the healthcare providers and with families [19].

FCC has been implemented in several high-income countries [20] including Sweden [7], Canada [9], Japan [21], United States [22], Italy [23], and Australia [24]. Outside of high-resource settings, the approach has not been as widely used, although pilot studies have been conducted in South Africa [25] and Iran [ 26] and a version of this approach is used in NICUs in Brazil [27]. Given the high burden of preterm births that occur in lower-middle-income countries (LMICs), the FCC approach has the potential to significantly improve neonatal outcomes in these settings [28]. There exists a need to document the FCC implementation process and examine the feasibility and acceptability of this model of care in low-resource settings.

In 2008, family engagement was introduced in the NICU at Ram Manohar Lohia (RML) Hospital, a busy tertiary hospital in New Delhi, to aid the nursing team in their duties and overcome severe workforce constraints that limit quality of care. This was the first implementation of family engagement in the NICU in India and the first time such a model has been tested in South Asia. Parents were trained on handwashing, entry protocol, breastfeeding, assisted feeding and skin-to-skin contact for eligible infants, developmentally supportive care, as well as recognition of danger signs and preparation for post-discharge care at home. In 2010–2012 a randomized controlled trial of the approach documented improved breastfeeding rates and no increase in nosocomial infections or adverse events [8].

Following the encouraging results of the trial, an FCC health education program was developed for the parent caring for a small or sick newborn in the NICU, linked to NICU care provider sensitization to effectively communicate with and support parents. This intervention is a paradigm shift in NICU care that transforms parent-attendant roles from mere passive observers to active willful participants with the care team through skills building and demonstrated competence. The program includes parent-attendant training on 1) handwashing skills; importance of infection prevention; protocol for entry to nursery; 2) developmentally supportive care (cleaning, sponging, positioning, nesting, handling and interacting with the infant; breastfeeding techniques, expression of breastmilk and assisted feeding); 3) kangaroo mother care; and 4) preparation for discharge and care at home. The resulting collaboration between the families and the healthcare providers creates a mutually respectful partnership that fosters communication between the two stakeholders and develops trusting relationships. In 2014, RML Hospital collaborated with the Norway India Partnership Initiative to pilot test the model in five district-level special newborn care units. Based on the study’s results, the Government of India issued a national policy to scale up FCC in all 700 district special newborn care units [29, 30]. In this study, we examine the feasibility and acceptability of implementing this model in the NICU. The results of this evaluation will be used to inform further development of the implementation framework for the FCC model and inform scale-up of this model within India and beyond.

## Methods

### Study site and characteristics

We conducted this study in the 14-bed NICU at RML Hospital in New Delhi, India. It is a referral neonatal unit in a teaching hospital which provides tertiary neonatal care including assisted ventilation and major surgery for sick newborns. NICU care at the study facility, as well as other facilities in the public health system, is absolutely free of cost. However there are private sector facilities in India that provide care at a cost levied to the patient and his/her family. During each shift, the unit is staffed by up to three nurses and two resident doctors (one senior and one junior). The NICU admits an average of 30 infants per month and has 2 sub-divisions: the intensive care area and a step-down care area for care to infants graduating from intensive care. A dedicated breastmilk expression and storage room with a refrigerator has been created in the unit. Kangaroo mother care (KMC) is provided by the bedside. All eligible infants admitted to the unit during the study period were recruited for participation in the study. Daily trainings for parent-attendants were provided in a designated area situated adjacent to the clinical area with seating for 12–15 persons and an LED television set.

### Study design and instruments

This feasibility and acceptability study was conducted using a prospective cohort design. The study protocol and data collection materials were developed by the study team at RML Hospital. We collected quantitative data on all study participants at enrollment, during the NICU stay, and at discharge using structured participant files.

### Study measures

We examined the feasibility and acceptability of implementing an FCC model using seven measures. Feasibility measures addressed whether interventions were implemented as intended, such as sensitizing sessions for all healthcare providers, training all parent-attendants, and frequency of training sessions. Acceptability measures focused on whether the intervention was acceptable to parent-attendants and healthcare providers as measured by whether healthcare providers conducted parent-attendant training sessions and whether parents were able to complete all activities as intended. Feasibility and acceptability measures are described in Table 1.

**Table 1:** Family-centered care feasibility and acceptability measures

Measure	Description	Population
**Feasibility Measures**		
Willingness of eligible parent-attendant/infant dyads to participate in the FCC program.	Measured as a percentage of total eligible dyads that agreed to participate in the study.	Parent-attendants
Frequency of parent-attendant training sessions per schedule.	Measured as a percentage of sessions that were held, out of the total number of planned training sessions.	Healthcare providers
NICU healthcare providers’ being sensitized and aware of the FCC program running in the unit.	Measured as the percentage of doctors and nurses sensitized, out of the total number of doctors and nurses on the unit. Data collected during four time points.	Healthcare providers
**Acceptability Measures**		
Parent-attendants’ attendance in training sessions.	Measured as the percentage of participating parent-attendants who completed all sessions, only sessions 1 and 2, only session 3, and only session 4.	Parent-attendants
Daily participation/engagement of parent-attendants in bedside activities of care of their infants.	Activities were monitored daily for all participants. Measured as the average daily completion for each activity during the study period.	Parent-attendants
Competence of the performance of parent-attendants’ activities.	Activities monitored by providers during first two months of program implementation. Measured as the percentage of parent-attendants completing each activity correctly.	Parent-attendants
Providers’ participation in training the parent-attendants.	Measured as the percentage of trained providers who conducted parent-attendant training sessions.	Healthcare providers

*Abbreviations: FCC* family-centered care, *NICU* newborn intensive care unit

### Ethical considerations

The study met international ethics requirements and was approved by the RML Hospital New Delhi Ethics Committee, RML Hospital, New Delhi, India, and Quorum Review IRB, Global Alliance to Prevent Prematurity and Stillbirth (GAPPS), Lynnwood, WA, USA (No. 33218). Verbal consent was approved by the Ethics Committee and was obtained from all parent-attendants and healthcare providers participating in the study prior to data collection. We obtained verbal consent in their preferred language to avoid any potential perceived intimidation by requesting a signature, and informed them that their participation would be voluntary and there would be no professional or personal consequences nor benefits of participation. Mothers were given the option to read or hear their consent form according to their literacy level. To avoid possible coercion, no financial incentives were provided.

### Inclusion criteria and sampling

Parent-attendant/infant dyads were eligible to participate in the study if the infant was hemodynamically stable (not on inotropes), not on ventilation (invasive or noninvasive), and the parent-attendant was willing and available to provide care to the infant. Exclusion criteria included the following: the infant was not hemodynamically stable, the infant was on ventilation, and the parent-attendant was unwilling or unavailable to provide care to the infant.

Of all 395 NICU admissions that occurred during the study period, 1 parent-attendant refused to participate, and 2 infants did not have an available parent-attendant and were therefore not eligible. Another 59 infants were found not to be eligible due to clinical exclusion criteria during their admission. Parents and family members of all eligible infants admitted to the NICU from June 2016 through July 2017 were included in this study.

Data were collected on three groups of participants: 1) parent-attendants, 2) infants, and 3) healthcare providers (doctors and nurses) working in the unit. For each infant admitted to the unit, we identified a primary parent-attendant (either mother, father, grandparent, or other family member), who would be available and willing to care for the infant during the study. Some infants were cared for by more than one parent-attendant; however, for the purpose of this analysis we considered data about the primary parent-attendant only. A total of 392 parent-attendant dyads/infant participated in the study. All healthcare providers assigned to the NICU during the study period were included.

### Data collection, management, and analysis

Data were collected from enrollment through discharge for each parent-attendant/infant dyad. Compliance of the parent-attendant with FCC program activities was directly observed during June and July 2016. All data collected from parent-attendant/infant dyads were recorded by a study nurse and entered into the study database. Data collected include demographics, parent-attendant training session attendance, parent-attendant activities, unit staff sensitization sessions attendance, and staff participation in conducting training sessions for parents. All data were de-identified and analyzed in Excel. Descriptive analyses were completed to summarize study sample characteristics for parent-attendants, healthcare providers, and newborns. All continuous variables were summarized with means, percentages, and ranges. Infants with missing data were excluded from the analysis. However, those who died or left the unit against medical advice, but had complete data recorded, were included in the analysis. Data on healthcare providers were obtained on a quarterly basis from the routine NICU records that did not record personal identifiers.

### Intervention characteristics and implementation model

**Fig. 1:**
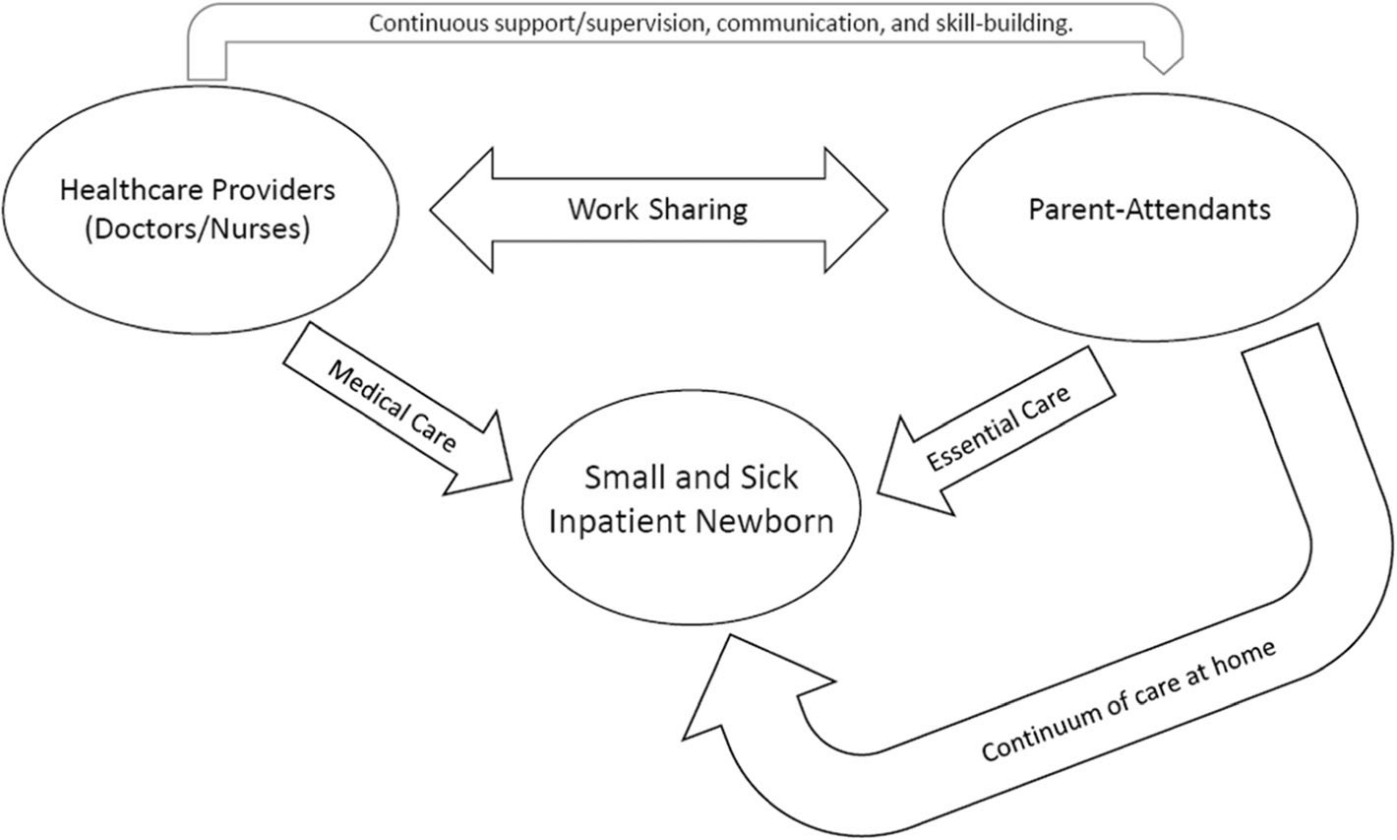
Operational concept for the implementation of family-centered care

The FCC intervention focuses on developing a participatory collaboration between healthcare providers and parent-attendants in the care of a sick newborn in the NICU. The FCC concept has been implemented in diverse ways, with a common focus on the infant, parent, and healthcare provider triad as a unit of care [31]. For the purpose of this study, we consider the parent-attendant and newborn dyad and the provider separately; however, all three are key participants in this model (see Fig. 1).

The healthcare provider component provided sensitization to the FCC approach and training of the healthcare providers as to how to train and support the parent-attendants in a consistent and standardized manner. Healthcare provider buy-in is essential to this model of care, and therefore sensitization sessions were carried out for healthcare providers at the beginning of the study period and every three months during the duration of the study. This accommodates the system of rotational postings from the pediatric department to the neonatal unit. Provider sensitization/training sessions were conducted by the principal investigator and included an in-person training session on the topics covered in the parent-attendant trainings and a discussion session. In addition to providing medical care to newborns, healthcare providers led skill-building training sessions for parent-attendants that focused on psychosocial and tangible support, and provided ongoing support, supervision, and continuous communication with parent-attendants in the NICU. Mothers were provided three meals daily at no cost. Financial support to families was provided through a hospital social worker in times of economic hardship. The hospital ensured that families did not have to incur any out of pocket expenditure for medical care that their baby received.

The parent-attendant training sessions were made available daily and the parent-attendants were granted autonomy to decide to participate or not, and could participate in sessions as often as they liked. A comprehensive audio-visual training tool with four sequential modules was developed with multidisciplinary technical input from a neonatologist, community medicine specialist, psychologist, nurse, and Hindi language expert. The topics included in each session are presented in Table 2. All new participating parent-attendants received training session 1 because it included the initial introduction to FCC and induction into the study. Sessions 1 and 2 were conducted daily as they covered the most essential topics and skills for all parent-attendants. Sessions 3 and 4 were conducted every other day as the former was only for a subset of infants who were eligible for KMC and the latter was only for infants who were expected to be discharged shortly.

**Table 2:** Content of family-center care training sessions for parent-attendants

Session 1: Sensitization to Family-Centered Care	• Describing the program • Preparing for entry into nursery (handwashing, gowning, infection prevention, familiarizing with nursery environment)
Session 2: Developmentally Supportive Care	• Minimizing noise, holding infant, nesting, calming infant • Cleaning of soiled infant • Breastfeeding • Expression of breastmilk • Spoon/cup feeding • Identifying danger signs and when to alert the healthcare provider
Session 3: Kangaroo Mother Care	• Upright positioning of infant • Providing skin-to-skin contact
Session 4: Preparation for Discharge and Care at Home	• Preparing for discharge and care at home • Handwashing/prevention of infection hygiene • Sponging/cleaning • Appropriate clothing/thermal care • Exclusive breastfeeding and skin-to-skin contact • Caring of cord and eyes • Identifying danger signs and when to seek medical care • Following up and complying with discharge instructions • Immunization

During the first two months of the study, nurses formally observed a subset of parent-attendants as they performed essential newborn care activities to assess uptake of the training topics and evaluate whether parents were able to correctly perform all activities. The nurse in charge of the observation randomly selected five parent-attendants for observation daily, observed all parent-attendant activities, and recorded compliance with proper protocol for all activities performed. During the remainder of the study period, nurses recorded whether each participating parent-attendant completed an activity during each day of admission but did not assess accuracy of the performed skills.

Fidelity of the training sessions for the parent-attendants and the sensitization sessions for the healthcare providers were assured through direct supervision from the senior nursing office in the NICU. There were no punitive measures or direct incentives provided for the healthcare providers to deliver the training sessions.

## Results

Of 395 NICU admissions during the study period, eligible participants included 333 parent-attendant/infant dyads, 24 doctors, and 21 nurses. Most parent-attendants were mothers (68%), between the ages of 20–35 (87.6%), and either illiterate or had completed secondary school (59.2%). Twenty percent of parent-attendants were fathers, and 12% were other family members. The average length of stay in the NICU was 15.8 days and the average age at admission was 8.9 days. Of all infants admitted to the NICU during the study period, only 35.5% were female and only 38% were exclusively breastfed (Table 3).

**Table 3:** Characteristics of NICU parent-attendants and newborns

Characteristics	Number^a^	Percentage
***Parent-Attendants, n = 444***		
Age (years)		
20–35	389	87.6
36–50	39	8.8
> 50	16	3.6
Sex		
Female	320	72.1
Male	124	27.9
Relationship		
Mother	302	68.0
Father	89	20.0
Other	53	12.0
Literacy level		
Illiterate	97	21.8
Secondary school	166	37.4
High school	70	15.8
Graduate	77	17.3
Postgraduate	34	7.7
***Newborns, n = 395***		
Sex		
Female	140	35.5
Male	255	64.5
Avg. length of stay (days)	15.8	–
Mean deaths per month	4	–
Mean birthweight (g)	2291	–
Mean age at admission (days)	8.9	–
Mean age at discharge (days)	24.4	–
Feeding		
Exclusively breastfed	–	38
Partial breastmilk	–	31
Formula	–	17

^a^Parent-attendant total includes all parents involved in family-centered care of the newborn

### Feasibility

Feasibility of the FCC program was measured by assessing whether the program training components were implemented as intended, and measuring the participation rates of healthcare providers in sensitization sessions. Because turnover of NICU healthcare providers was routine, sensitization sessions were held four times during the study period, in December 2016, June 2016, March 2017, and July 2017. Healthcare providers who received the sensitization then had the option to conduct parent-attendant trainings.

**Fig. 2:**
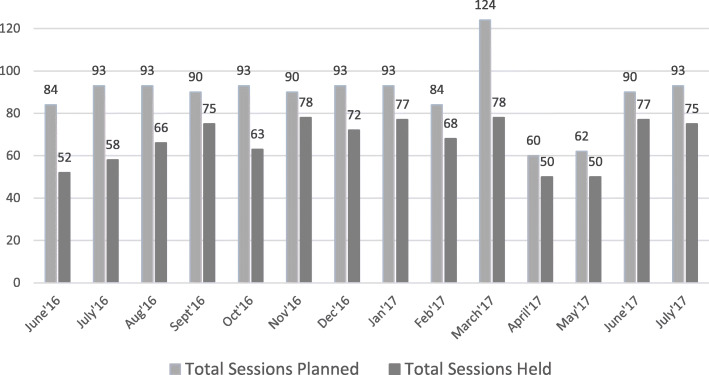
Number of family-centered care parent-attendant training sessions planned and held during the study period

Of the 1242 planned parent-attendant training sessions, 939 (75.6%) were held, indicating that program fidelity was high, and most trainings were implemented as intended (Fig. 2).

The percentage of doctors and nurses in the unit that received FCC sensitization training ranged from 50% (21/42) to 84% (36/44). The percentage of healthcare providers receiving FCC training increased in the second half of the study, with more than 83% of staff trained in 2017. Provider training data were collected during discrete time points, therefore multiple provider training contacts are possible.

### Acceptability

The FCC program was acceptable to both parent-attendants and healthcare providers. Parent-attendant acceptability was measured by the percentage of parents who participated in the program trainings and the parent-attendants’ ability to accurately complete program activities. Of the 395 parent-attendant/infant dyads admitted to the NICU, 392 (99.2%) agreed to participate in the FCC program and had at least 1 parent-attendant available. One parent refused participation and two infants did not have an available parent-attendant and were therefore not eligible.

Parents and family members were willing to participate in trainings and provide essential care to infants in the NICU. Training session 1 was conducted during study enrollment, and therefore all participating parent-attendants completed training session 1. While only 50% of parent-attendants (167/333) completed all 4 FCC training sessions, 95% (316/333) completed sessions 1 and 2, required for all participants; 75% (249/333) completed session 4 which covered preparation for discharge and care at home; and 60% of the total participating parent-attendants (200/333) completed session 3 which focused on KMC. Most parent-attendants were able to participate in the care of their infants in the NICU by completing the specified FCC activities daily throughout the study period.

**Fig. 3:**
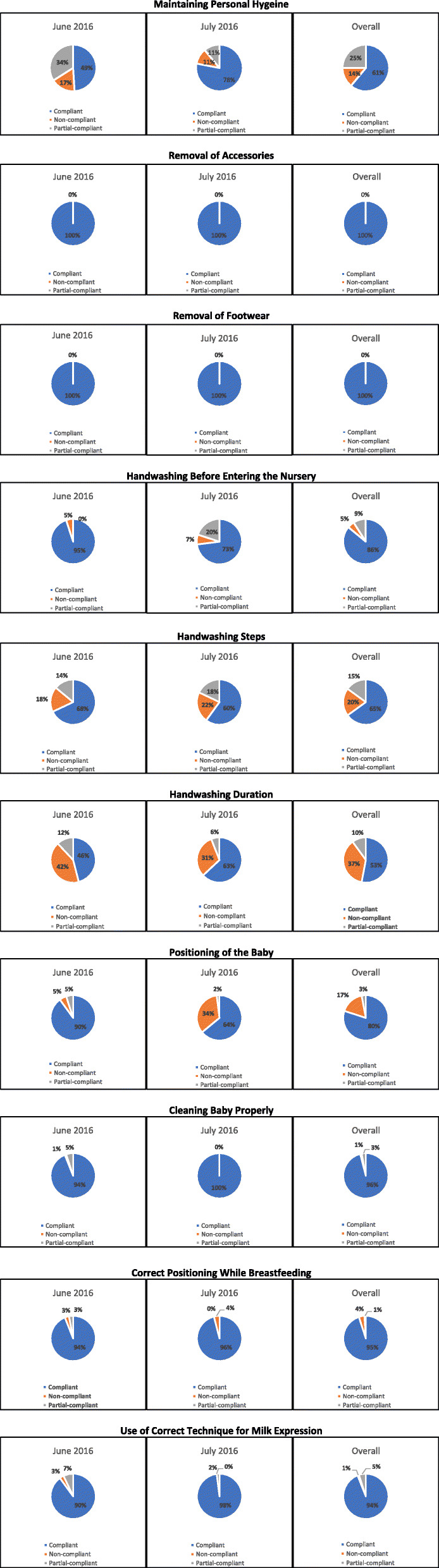
Observed parent-attendant compliance with family-centered care program activities during June 2016, July 2016, and overall (*n* = 333)

Observed compliance rates were over 96% for 5 of 10 FCC parent-attendant activities, and 60 to 78% for the remaining 5 activities. (Fig. 3) The newborn care activities that were monitored were positioning (74% compliance) and cleaning of a soiled infant (80% compliance). During the first two months of FCC implementation, parent-attendant compliance with key program activities was directly observed by staff nurses (Fig. 3). Rates of compliance of the 10 essential activities varied widely, with some activities showing 100% compliance in both months (removal of accessories and removal of footwear), while others showed improvement from June 2016 to July 2016 (personal hygiene, handwashing duration, cleaning infant, and use of correct breastmilk expression technique). Notably, several activities showed a decrease in compliance, including handwashing prior to entering the NICU, following handwashing steps, and proper positioning of the infant.

**Fig. 4:**
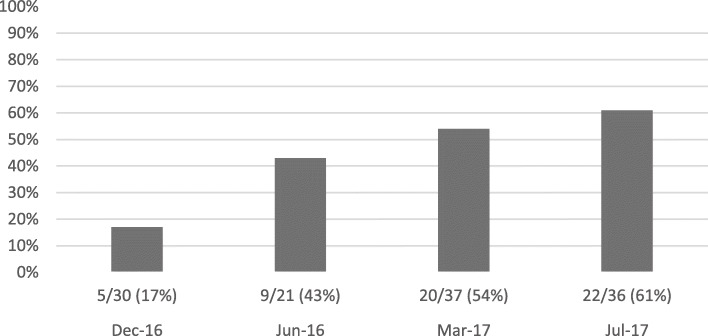
Number and percentage of healthcare providers participating in conducting parent-attendant family-centered care training sessions

Acceptability of the FCC intervention to healthcare providers was measured by the percentage of all participating healthcare providers who participated in conducting parent-attendant training sessions after being sensitized to the program. Provider data were collected on a quarterly basis. The percentage of healthcare providers conducting parent-attendant trainings increased steadily during the study period, with more than 50% of healthcare providers participating in training parent-attendants during 2017 (Fig. 4).

## Discussion

Of the 4 million newborn deaths each year, 99% are in low- and middle-income countries [32]. In India, neonatal deaths make up three-quarters of infant deaths and almost half of those deaths occur in the first two days of life [33]. Up to 50% of these deaths could be prevented by implementing health facility-based interventions [34]. Facility-based newborn care is therefore a key strategy for improving survival of newborns in LMICs [4]. The current state of NICU care in lower-income countries presents challenges in ensuring children are not separated from their parents against their will. This formative study describes the implementation, feasibility, and acceptability of an FCC model implemented in an urban NICU at a tertiary hospital in New Delhi, India. We found that FCC was feasible to implement in this setting and the program was acceptable to participating parents, family members, and healthcare providers.

The family visiting policy in the NICU was favorable at the study facility. The hospital policy at the time of the study allowed either the mother or father to be present in the NICU without time restrictions, but was limited to one person at a time to avoid overcrowding. In the uncommon instance when the mother or father were not available, an alternate attendant from the family was allowed. Siblings were not allowed.

The hospital philosophy and strategic priorities relating to FCC improved gradually as evidence was generated that an FCC approach when practiced in the NICU does no harm and is beneficial to newborns. The hospital leadership has acknowledged that the FCC approach to NICU care improves newborn outcomes, increases parent satisfaction, and is consistent with ethical principles for the provision of medical care.

Healthcare provider sensitization and buy-in were key to driving implementation and participation in the FCC intervention. Through iterative learning during implementation we learnt that training only parent-attendants was not sufficient. Parents were already onboard, waiting to be involved in their own baby’s care in NICU. The bigger challenge was overcoming the conventional mindset of healthcare providers that assume a position of commanding authority in the traditional provider-centered care model. Psychosocial and tangible support and education offered to participating family members depends strongly on engaged healthcare providers [35, 36]. Healthcare providers played an important role in this program and their buy-in was essential for skill-building and engagement of parents and family members. Acceptance of the program improved over time, as shown by the steady increase in the proportion of healthcare providers who conducted parent-attendant training sessions throughout the study. The proportion of healthcare providers who received sensitization training did not increase significantly in the second half of the study period. We attribute this increase in healthcare providers that conducted parent-attendant trainings to greater staff familiarity and confidence in the FCC program that led to increased acceptance and participation by healthcare providers.

Recent studies of FCC implementation have found that this approach results in increased provider satisfaction and job performance, although the immediate impact of the intervention has been found to present challenges for nurses and healthcare providers, such as increased time away from patient care, difficulty in coordinating communication, and increased time spent on supporting and communicating with parents [18, 37–39]. While FCC is intended to decrease provider work burden through work sharing with parents and family members, this study did not specifically assess the impact of this intervention on provider satisfaction, stress levels, and burnout. Our results indicate that healthcare providers found this intervention acceptable and feasible to conduct; however, more research is needed to assess the impact of FCC on healthcare providers in this setting.

Most research on the implementation of FCC models has focused on the experience of and benefits to parents and family members participating in the intervention [31]. In the present study, parents found FCC acceptable and feasible as shown by the high participation rates, high rates of training completion, and high compliance with program activities. Parent and family member participation was highest in the first two training sessions of the program. These sessions were mandatory for participants and study findings indicate that almost all participants completed these trainings. Although session 4 was not mandatory, it covered content preparing participating parents and family members to care for infants at home after discharge from the unit and presents a key part of the FCC model. One of the goals of FCC is to build parent and family skills in caring for small and sick newborns during inpatient care and to carry these skills on to care at home to maintain a continuum of care. The final training session is a key part of the FCC model and has the potential to impact long-term health outcomes for newborns. A more in-depth study of the impact of FCC on parent caregiving skills post-discharge and the impact of this on health outcomes is needed.

Early adoption and sustained demonstration of desired activities is an indication of acceptability of the intervention and impact on behaviors. The overall compliance rates for the FCC activities were over 90%. Low compliance rates for particular FCC activities suggest either greater challenges for parents and family members or lower acceptability of these activities. Compliance with handwashing protocols was lower during the second month of observation than at the beginning of the study period, suggesting that this element may be less acceptable to parents and may make sustained implementation of this intervention challenging. Although not the primary outcome of this study, the decrease in compliance with certain activities may indicate parent-attendant fatigue, provider fatigue, or a particularly challenging activity for parents.

Given the required strong focus on handwashing and infection prevention for newborns in hospital settings, the relatively lower compliance with handwashing is an important finding of this evaluation. While the benefits of infection prevention including hand hygiene are significant and demonstrable [40], achieving high rates of compliance by parent caring for newborns remains an obstinate challenge for many hospitals and health systems. Data on hand hygiene practices is lacking for parents caring for newborns in hospital settings. Estimates from various hospital settings for healthcare workers report initiation of hand hygiene less than 50% of the time they should [41]. Additional research is needed to examine the challenges of hand hygiene protocols for parents caring for inpatient small and sick newborns in this setting and whether higher compliance rates are achievable. Low compliance may be a result of low acceptability or the challenge of integrating several new skills over a short period of time for participating parents and family members.

Efforts to sustain the fidelity of the FCC intervention were focused on the healthcare providers. The sensitization sessions and training directed to provide psychosocial and tangible support to parent-attendants led by experienced healthcare providers were scheduled regularly as new staff rotate into the NICU. A roster of trained healthcare providers was maintained and these providers were routinely scheduled for the daily parent-attendant trainings. The senior nursing officer in the NICU oversaw all FCC sensitization and training activities with support from the NICU medical director.

During the onset of the COVID-19 pandemic in March 2020, practice of FCC in the NICU had a setback. Parent-attendant entry to the NICU was restricted until symptom screening and testing became available. Implementation of FCC has been maintained with appropriate COVID-19 transmission prevention measures in place. There remains an indispensable need for FCC, including the infection prevention training component.

This model of FCC is currently being scaled in India at sites across the country, which will lead to additional learning about the implementation process and the feasibility and acceptability of this approach within different healthcare settings in India and other contexts. Initial findings show that to date, 85 districts have implemented FCC, reaching over 13,000 mothers and family members. Eighty-six percent of newborns below 2000 g received KMC and exclusive breastfeeding; 75% continued to benefit from KMC at home; and post-discharge mortality was reduced from 7 to 3% [29]. The present evaluation offers several opportunities for further study, including the need for a more in-depth assessment of the impact of FCC on newborn health outcomes and post-discharge care, as well as additional study of the impact of FCC on healthcare providers. In addition, documenting the implementation experience and lessons learned across a variety of healthcare settings and geographies would offer valuable tools to help other LMICs in designing implementation models tailored to their context [31]. These efforts are especially timely as this model of FCC creates a paradigm shift in the hospital treatment of sick newborns in India and potentially other LMICs that ensures a child is not separated from his or her parents against their will.

### Limitations

The generalizability of the findings to other Indian and international contexts has limitations. This intervention was implemented in an urban tertiary level NICU at a publicly supported hospital in New Delhi, and therefore these results may not be representative of all neonatal care facilities in India, especially in rural settings, or at private fee-for-service hospitals. Based on a 2011 review of NICU function, use, and staffing in eight Indian states [4], the NICU in the current study is mid-sized, and infants participating had a longer median stay compared with that of other Indian NICUs. The transferability of these results may in some part depend on the availability of prerequisite infrastructure, such as conference rooms to provide educational training sessions and adequate number of NICU beds for single occupancy. Overall, the high levels of feasibility and acceptability of the FCC approach by parent-attendants and healthcare providers suggests that introduction of the model with local adaptation of the training materials for parent-attendants and healthcare providers and small infrastructure investments is warranted in settings with appropriate monitoring.

This study used direct observation of parent-attendant compliance with key activities, which was conducted only during the first 2 months of implementation; therefore, this may reflect a wash-out period effect as parent-attendants and healthcare providers became sensitized to the intervention and more familiar with program activities and concepts. In addition, while this study allowed for infants to be cared for by multiple parents or family members, complete data were collected only for the primary parent-attendant. This may have resulted in continuity issues in the care of the infant, and the data used in the present analysis may therefore not be fully representative of all caregivers who participated in the study. Finally, this evaluation was limited to quantitative data and analyses on acceptability and feasibility; a qualitative assessment of this model is published separately [42]. Therefore, some of the nuance and context around the implementation of the FCC intervention may not be captured in this evaluation.

## Conclusions

FCC was feasible to implement in the NICU setting and the program was acceptable to participating parent-attendants and healthcare providers. Parents participated in trainings conducted by NICU providers and engaged in essential care to their infants on a day-to-day basis in the NICU. The FCC program establishes a standard care approach and behavior norms for healthcare providers that direct psychosocial and tangible support to parent-attendants so that a child is not separated from his or her parents against their will while receiving advanced care in the NICU. Expanding this intervention with monitoring across a range of NICUs, including in more rural settings, will be key to inform scaling of the FCC model in India and other LMICs.

## Data Availability

The datasets used and analyzed during the current study are available from the corresponding author on reasonable request.

## Abbreviations

FCC: Family-centered care
KMC: Kangaroo mother care
LMICs: Lower-middle-income countries
NICU: Neonatal intensive care unit
RML: Ram Manohar Lohia
